# Multiple roles of ALDH1 in health and disease

**DOI:** 10.3389/fphys.2025.1627164

**Published:** 2025-07-10

**Authors:** Wenqi Zhao, Yu Xia, Zhiqi Gao, Jian Chen, Erlong Zhang

**Affiliations:** ^1^ Institute of Medicine and Equipment for High Altitude Region, College of High Altitude Military Medicine, Army Medical University, Chongqing, China; ^2^ Key Laboratory of Extreme Environmental Medicine, Ministry of Education, Chongqing, China

**Keywords:** aldehyde dehydrogenase, ALDH1, cancer, molecular regulation, stem cells marker

## Abstract

Aldehyde dehydrogenase 1 (ALDH1) is an important aldehyde-metabolizing enzyme that plays a key role in various biological processes, such as regulating cellular growth, modulating retinoic acid (RA) signaling pathways, and contributing to stem cell function. It is recognized as a crucial biomarker not only because of its significant involvement in the occurrence and development of various cancers but also because it is an important prognostic indicator for cancer progression. Furthermore, recent research has provided substantial evidence that the multiple roles of ALDH1 extend beyond cancer, with significant progress in understanding its mechanisms in other diseases and its clinical applications. These findings provide potential therapeutic targets for the future treatment of other diseases. In this review, we summarize the current understanding of the biological functions of ALDH1, the molecular mechanisms of its transcription and regulation, and the progress in cancer research related to the ALDH1 family. We not only discuss the mechanisms of ALDH1 in cancer but also its research development and potential pathological mechanisms in other diseases. The role of ALDH1 in various diseases is complex, and its expression levels are highly important for disease diagnosis, treatment, and prognosis. Future in-depth studies on ALDH1 functions are expected to provide new strategies and directions for the treatment of related diseases.

## 1 Introduction

The aldehyde dehydrogenase (ALDH) superfamily comprises a class of NAD(P)^+^-dependent multifunctional enzymes consisting of nineteen isozymes categorized into 11 families and 4 subfamilies (Vassalli, 2019). These enzymes catalyze the oxidation of both endogenous and exogenous aldehydes into their corresponding carboxylic acids, thereby protecting organisms from aldehyde-induced toxicity. ALDH1 represents one of the critical subgroups within this superfamily that encompasses six family members (ALDH1A1, ALDH1A2, ALDH1A3, ALDH1B1, ALDH1L1, and ALDH1L2), located primarily on chromosome 9q21 (Wang et al., 2020). Notably, research has suggested that, compared with other ALDH enzymes, ALDH1 may possess a more significant functional repertoire.

The ALDH1 family primarily localizes to the cytoplasm of hepatocytes and performs critical physiological and pathological functions in humans. In addition to its fundamental role in acetaldehyde detoxification, it mediates essential processes, including retinoic acid (RA) synthesis and lipid metabolism, and serves as a stem cell marker (Duan et al., 2024; Nguyen et al., 2024). RA, a vitamin A/retinol derivative, functions as a pivotal signaling molecule with profound implications for health and disease. As a well-established developmental morphogen, it primarily acts through the transcriptional activity of nuclear RA receptors (RARs) (Wu et al., 2024). Research has confirmed that ALDH1A2 (also known as retinaldehyde dehydrogenase 2 (ALDH2)) is the dominant enzyme during embryogenesis. Postsynthesis, RA acts as a ligand for nuclear RARs, which form heterodimers with retinoid X receptors (RXRs). These RAR/RXR heterodimers bind to RA response elements and recruit transcriptional coactivators upon ligand binding, initiating the transcription of target genes that govern proliferation, differentiation, and apoptosis (Leon et al., 2023). In lipid metabolism, intracellular lipases catalyze triglyceride hydrolysis into glycerol and free fatty acids, playing pivotal roles in lipid processing. Studies have confirmed that ALDH1A1 gene overexpression and RNA interference promote and inhibit lipid accumulation and triglyceride production in mature adipocytes, respectively, as well as the expression of LPL and its transcription factors (PPARγ and C/EBPα) (Hu et al., 2021). Therefore, as a cellular lipase, ALDH1 proteins not only oxidize retinal and fatty aldehydes in different cytoplasmic compartments but also play crucial roles in gene expression and tissue differentiation across various tissues (Wang et al., 2020; Wei et al., 2022).

In recent years, numerous studies have confirmed that ALDH1 expression can serve as a stem cell marker for various types of tumors (Wei et al., 2022). ALDH1 is significantly overexpressed in solid tumors such as those seen in patients with colorectal cancer, lung cancer, and breast cancer (Wang et al., 2022; Feng et al., 2022; Bush, 1989). ALDH1 has thus become a potential therapeutic target for cancer stem cells in solid tumors, providing new targets and a foundation for the treatment of these cancers (Duan et al., 2024). Furthermore, many studies have shown that ALDH1 family members not only serve as markers for tumor stem cells but also catalyze the conversion of retinaldehyde to retinoic acid, thereby regulating cell differentiation (MacDonagh et al., 2021). The ALDH1 family can therefore influence tumor initiation and progression through the RA signaling pathway through its ability to induce differentiation via RA. In addition, ALDH1 can further detoxify aldehydes produced by oxidative stress to protect cells from oxidative stress and damage (Calleja et al., 2021) and inhibit the production of reactive oxygen species (ROS), thereby reducing DNA damage and cell apoptosis (Poturnajova et al., 2021). Thus, this review mainly discusses the functional mechanisms of the ALDH1 family and its role in human diseases, as well as the impact of its various regulatory actions on disease outcomes.

## 2 The functions of the ALDH1 family

### 2.1 ALDH1 in alcohol metabolism and aldehyde detoxification

**FIGURE 1:**
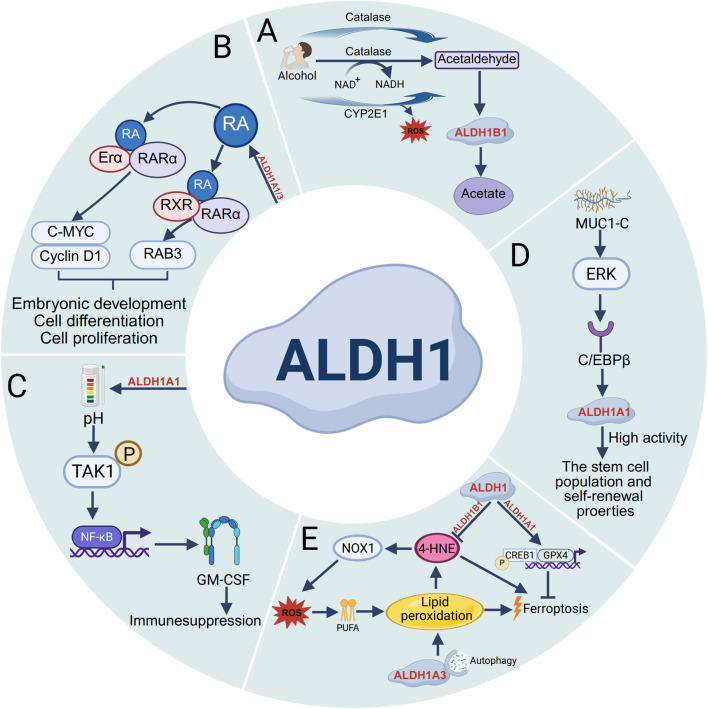
Functions of the ALDH1 family. **(A)** In alcohol metabolism, ethanol undergoes oxidation catalyzed by enzymes such as alcohol dehydrogenase (ADH), cytochrome P450 2E1 (CYP2E1), and catalase into acetaldehyde. CYP2E1 promotes the production of acetaldehyde as well as the formation of ROS. Acetaldehyde is then converted to acetate by ALDH1B1. **(B)** The ALDH1 family modulates RA binding to ERα and RAR, thereby stimulating the transcription of Cyclin D1 and c-MYC. Additionally, it regulates RA-induced binding of RARα and RXR to initiate RARβ transcription, Ultimately affecting the processes of embryonic development, cell differentiation, and proliferation. **(C)** The expression of ALDH1A1 reduces intracellular pH and promotes the phosphorylation of transforming growth factor β (TGFβ)-activated kinase 1 (TAK1), thereby activating nuclear factor κB (NF-κB) signaling and increasing the secretion of granulocyte–macrophage colony-stimulating factor (GM-CSF), ultimately leading to decreased T-cell immunity and immune suppression. **(D)** The transmembrane C-terminal subunit of Mucin 1 (MUC1) is aberrantly overexpressed in most human breast cancers, which signals through extracellular regulated protein kinase (ERK) and promotes the high activity of ALDH1A1 after stimulation through the transcription factor C/EBPβ, promoting stem cell population self-renewal properties. **(E)** During ferroptosis, ALDH1B1 directly metabolizes lipid peroxidation-derived 4-hydroxynonenal (4-HNE) to suppress its accumulation. ALDH1A3 regulates ferroptotic progression through autophagy, while ALDH1A1 activates both glutathione peroxidase 4 (GPX4) and cAMP-responsive element-binding protein 1 (CREB1) to modulate ferroptosis.

The ALDH1 enzyme family plays a crucial role in intracellular metabolism, particularly in the key reaction of catalyzing the oxidation of acetaldehyde to acetate. In the alcohol metabolism pathway, ethanol is first converted to acetaldehyde by alcohol dehydrogenase (ADH), cytochrome P450 2E1 (CYP2E1), and catalase. Subsequently, acetaldehyde is metabolized primarily to acetate by mitochondrial ALDH1B1 (Hyun et al., 2021). (Figure 1A). And ALDH1 family protects and detoxifies the body by exercising its dehydrogenase activity to oxidize aldehydes into carboxylic acids. After oxidative stress damages DNA and proteins, it triggers lipid peroxidation of cellular phospholipids, producing more than 200 types of reactive aldehydes. ALDH1A1 and ALDH1A3 detoxify and protect cells from these and their use has become a common detoxification strategy in cancer treatment (Poturnajova et al., 2021).

### 2.2 ALDH1 regulates the RA signaling pathway

The ALDH1 enzyme family plays a pivotal role in retinol metabolism, serving dual functions: (1) mediating the reversible conversion between retinol and retinaldehyde and (2) catalyzing the irreversible oxidation of retinaldehyde to retinoic acid (RA) in retinal and other tissues. As a crucial signaling molecule, RA exerts its biological effects by binding to the nuclear receptors RAR/RXR, thereby regulating downstream gene expression networks that govern critical cellular processes, including differentiation, proliferation, and fate determination (Toledo-Guzman et al., 2019). As shown in Figure 1, studies have revealed distinct expression patterns of ALDH1A1 and ALDH1A3 in prostate cancer tissues that correlate with clinical outcomes. ALDH1A1 promotes tumor progression by enhancing the transcriptional activity of both the androgen receptor (AR) and the retinoic acid receptor (RAR), whereas ALDH1A3 appears to exert inhibitory effects. Further investigation has revealed PLK3 as a target gene that is reciprocally regulated by ALDH1A1 and ALDH1A3 in a RAR/AR-dependent manner (Gorodetska et al., 2024). Moreover, in osimertinib-resistant cancer cells, S100A9 was found to activate the RA signaling pathway by upregulating ALDH1A1 expression, thereby promoting brain metastasis. Notably, genetic inhibition of S100A9 or ALDH1A1 or pharmacological blockade of RA receptors (RARs) significantly reduces metastatic foci formation, highlighting the therapeutic potential of targeting this pathway (Biswas et al., 2022) (Figure 1B).

### 2.3 Functional role of ALDH1 in stem cells

In various cancers, the expression level of ALDH1 is correlated with the characteristics of cancer stem cells, which play crucial roles in tumor initiation and progression. For example, ALDH1A3 is the most highly expressed ALDH gene in normal human breast tissue stem cells, whereas ALDH1A1 is expressed at low levels in all breast epithelial cells (Vassalli, 2019). Current research has demonstrate that ALDH1A1 enzymatic activity facilitates breast tumor growth. Mechanistically, ALDH1A1 decreased the intracellular pH in breast cancer cells to promote phosphorylation of TAK1, activate NF-κB signaling, and increase the secretion of GM-CSF, which led to myeloid-derived suppressor cell expansion and immunosuppression (Liu et al., 2021) (Figure 1C). Mucin 1 (MUC1) is a heterodimeric protein that is aberrantly overexpressed in most human breast cancers. Studies have confirmed that MUC1-C induces ERK signaling and thereby phosphorylation and activation of C/EBPβ, and show that a complex of MUC1-C and C/EBPβ occupies the ALDH1A1 promoter and induces ALDH1A1 expression, promotes breast cancer progression (Poturnajova et al., 2021) (Figure 1D). Additionally, studies have shown that ALDH1A3 is highly expressed in heart-derived progenitor cells. ALDH1A3 expression was confirmed at the protein level, and inhibition of ALDH1A3 expression using specific siRNAs significantly reduced ALDH activity and cell proliferation (Puttini et al., 2018). Many studies on cancer stem cells have demonstrated significant differential expression of ALDH1 across patients with cancers such as gastric cancer, colorectal cancer, cervical cancer, and ovarian cancer. Therefore, using ALDH1 expression and activity is an effective method for identifying and assessing cancer stem cells. Not only can ALDH1 be used as a marker, but cells with high ALDH1 expression typically exhibit increased proliferation, invasion, and migration abilities and drug resistance. In the future, ALDH1 could be considered a potential therapeutic target for cancer treatment.

### 2.4 ALDH1 in lipid peroxidation and ferroptosis

Ferroptosis is an iron-dependent, non-apoptotic form of programmed cell death, characterized primarily by lipid peroxidation resulting from the excessive accumulation of intracellular lipid reactive oxygen species (ROS) (Dai et al., 2024). Lipid peroxidation generates diverse oxidation products, with aldehydes serving as the predominant end products. Notably, ALDH1B1 can metabolize the aldehyde substrate 4-hydroxynonenal (4-HNE) at high concentrations. Supraphysiological levels of 4HNE trigger ferroptosis, whereas lower concentrations enhance cellular susceptibility to classical ferroptosis inducers by activating the NOX1 pathway (Chen et al., 2022). Furthermore, ALDH inhibition completely blocks the RSL3-dependent binding of ALDH1A3 to LC3B and the subsequent autophagic degradation of ferritin (ferritinophagy), indicating that that ALDH1A3 contributes to ferroptosis by mediating iron release via ferritinophagy (Wu et al., 2022). Additionally, ALDH1A1 activates the CREB1/GPX4 pathway, stimulates pH-dependent lipid droplet production, and thereby protects against KRAS inhibitor-induced ferroptosis (Bian et al., 2024). Collectively, these results provide valuable insights into targeting ALDH1 to enhance the efficacy of ferroptosis-based cancer therapies (Figure 1E).

## 3 Role of the ALDH1 family in cancer

Cancer, one of the leading causes of death worldwide, presents significant challenges related to research and treatment because of its complexity and heterogeneity. In recent years, research into the theory of cancer stem cells has provided new perspectives on cancer treatment. The ALDH1 family of important metabolic enzymes is closely associated with various cancer stem cells and plays different roles in different types of cancer (Table 1).

**TABLE 1:** Genetic and subcellular locations and physiological and pathophysiological roles of the ALDH1 family in various diseases. (The information in the table comes from https://www.proteinatlas.org and https://www.ncbi.nlm.nih.gov).

Isoenzymes	Chromosomal localization	Subcellular location	Distribution of major organs	Related diseases	Prognostic marker
ALDH1A1	9q21.13	Cytosol	Kidneys, lungs, stomach, liver, uterus, ovaries	Alcoholism, diabetes, neuronal development	Cervical squamous cell carcinoma,endocervical adenocarcinoma, Kidney renal clear cell carcinoma
ALDH1A2	15q21.3	Cytosol	kidneys, lungs, prostate, uterus	Arthritis of the hand, stomach cancer	Ovary serous cystadenocarcinoma
ALDH1A3	15q26.3	Cytosol	Prostate, small intestine, salivary glands	Breast cancer, anophthalmia, microphthalmia	Colon adenocarcinoma, Liver hepatocellular carcinoma, Ovary serous cystadeno-carcinoma
ALDH1B1	9p13.1	Mitochondria	Kidney, liver, prostate, skeletal muscle, stomach	Colorectal cancer, pancreatic cancer	Kidney renal clear cell carcinoma, Pancreatic adenocarcinoma, Thyroid carcinoma
ALDH1L1	3q21.3	Cytosol	Liver, kidney, brain, salivary glands, skeletal muscle	Non-small cell lung cancer, gastric cancer, neural tube malformations	Kidney renal clear cell carcinoma, Thyroid carcinoma
ALDH1L2	12q23.3	Mitochondria	Salivary glands, prostate, lungs, stomach, brain, uterus	Neurodevelopmental disorders, breast cancer	Ovary serous cystadenocarcinoma

### 3.1 ALDH1 as a cancer stem cell marker

Eradicating cancer stem cells (CSCs), often referred to as the “driving force” behind various malignant tumors, is crucial in cancer treatment (Sudhalkar et al., 2019). Since the proportion of CSCs within tumor tissues is relatively low, conventional cancer therapies often struggle to eliminate them completely, leading to tumor recurrence and metastasis. ALDH1 expression has now been widely confirmed as a marker for stem cells in various cancers, enabling the effective identification and sorting of CSCs, offering the potential for more precise future cancer treatments (Toledo-Guzman et al., 2019). For example, studies have shown that ALDH1, through its ability to oxidize retinol to retinoic acid, plays a role in early stem cell differentiation and is a strong candidate marker for breast cancer stem cells (Panigoro et al., 2020; Chen et al., 2020). Additionally, ALDH1A1 expression serves as a marker to identify a specific subpopulation of cancer stem cells in pancreatic ductal adenocarcinoma (Nimmakayala et al., 2021) Maryam Rezaee and colleagues reported that the expression of ALDH1, a CSC marker, is significantly elevated in tissues from patients with colorectal cancer (Rezaee et al., 2021). Furthermore, a study by EA Kogan revealed that in patients with COVID-19-related lung adenocarcinoma, the expression of ALDH1 was also significantly increased (Kogan et al., 2024). Recently, the use of ALDH1 activity as a marker has led to the successful isolation of CSCs from various tumor tissues, which is highly important for studying the biological characteristics of CSCs and developing new therapeutic strategies.

### 3.2 ALDH1 family involvement in cancer initiation and progression

The ALDH1 family is a key group of enzymes involved in cellular metabolism. High expression of ALDH1 enhances the self-renewal capacity, drug resistance, proliferation, and migration abilities of tumor cells. These characteristics enable ALDH1 family members to play important roles in tumor initiation, progression, metastasis, and recurrence, thus contributing significantly to tumor development. In studies on breast cancer, ALDH1A1 expression has been shown to lower the intracellular pH of breast cancer cells, which in turn promotes the phosphorylation of TAK1, activates the NF-κB signaling pathway, and results in the expansion of myeloid-derived suppressor cells and immune suppression, confirming that the enzymatic activity of ALDH1A1 promotes breast tumor growth (Liu et al., 2021). According to colorectal cancer research, tryptophan metabolites derived from anaerobic bacteria, such as trans-3-indoleacrylic (IDA), act as endogenous ligands for the aryl hydrocarbon receptor (AHR), leading to the transcriptional upregulation of ALDH1A3 expression. Loss of AHR or ALDH1A3 expression eliminates the tumor development promoted by IDA both *in vitro* and *in vivo*. These findings confirm that targeting the IDA-AHR-ALDH1A3 axis could be a potential therapeutic strategy for colorectal cancer related to ferroptosis (Cui et al., 2024). In addition, in EGFR-mutant lung cancer, S100A9 upregulates the expression of ALDH1A1 and activates the RA signaling pathway. Studies have confirmed that genetic inhibition of S100A9, ALDH1A1, or RA receptor (RAR) expression or treatment with pan-RAR antagonists significantly reduces brain metastasis. Therefore, the S100A9-ALDH1A1-RA pathway plays a key role in drug resistance and brain metastasis (Biswas et al., 2022). Qu et al. reported that the expression of ALDH1L1 was significantly reduced in patients with oral squamous cell carcinoma (OSCC) and that its downregulation was associated with tumor malignancy. They confirmed that upregulating ALDH1L1 expression can inhibit OSCC cell growth by inactivating the PI3K/Akt/Rb signaling pathway and slowing the progression of OSCC (Qu et al., 2023). In another study, ALDH1A3 was found to be highly expressed in high-grade serous ovarian cancer (HGSOC) tissues, promoting cell proliferation and drug resistance in HGSOC. Furthermore, ALDH1A3 can activate acetylated H3K27 to promote the expression of PITX1, thereby influencing the tumorigenesis of HGSOC (Huang et al., 2024). Therefore, the ALDH1 family provides new possibilities for disease treatment, and both its upstream and downstream pathways can serve as promising targets for therapeutic intervention.

### 3.3 ALDH1 expression is closely associated with cancer prognosis

ALDH1 is a CSC marker, and its expression levels in tissues of various malignancies are closely related to patient clinical prognosis. For example, in the progression of type I endometrial cancer (EC), the expression of cytoplasmic ALDH1 gradually increases among endometrial hyperplasia, atypical hyperplasia, and endometrial carcinoma. ALDH1 expression has been shown to be an early predictive factor for EC development, indicating that it can serve as an independent prognostic marker for the progression of endometrial hyperplasia, with or without atypia, to cancer (Mah et al., 2021). Sonar Soni Panigoro and colleagues reported that ALDH1 expression can be used as a prognostic factor for poor survival in patients with triple-negative breast cancer (TNBC) (Panigoro et al., 2020). Additionally, studies have shown that strong immunohistochemical expression of ALDH1 in OSCC subtypes may help identify patients with OSCC subtypes with poor prognosis (de Freitas Filho et al., 2021). Furthermore, research on ALDH1 expression in lung adenocarcinoma revealed that ALDH1 expression is positively correlated with epithelial-like phenotype proteins, and through immunohistochemistry and mRNA expression profiling of adenocarcinoma tissues, it was confirmed that ALDH1 expression is associated with good prognosis (Koh et al., 2019). Thus, ALDH1 expression is an independent favorable prognostic marker for overall survival or recurrence-free survival in patients with adenocarcinoma. These studies collectively demonstrate that expression of members of the ALDH1 family can serve as a predictive factor for poor prognosis in cancers, including type I endometrial cancer, triple-negative breast cancer, and OSCC.

## 4 ALDH1 family-related diseases

### 4.1 ALDH1 and Parkinson’s disease (PD)

**FIGURE 2:**
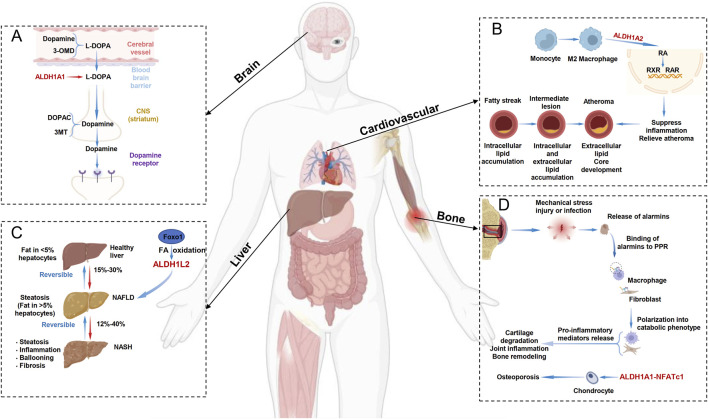
ALDH1 Family-Related Diseases. **(A)** In the dopamine metabolism process in the brain, ALDH1A1 converts the toxic substance L-DOPA into dopamine and can also convert it into the less harmful 3,4-dihydroxyphenylacetic acid (DOPAC). **(B)** In the progression of atherosclerosis, M2 macrophages can activate the RXR and RAR signaling pathways through ALDH1A2, thereby suppressing inflammation and slowing the progression of atherosclerosis. **(C)** In the development of non-alcoholic fatty liver disease (NAFLD), hepatocyte Foxo1 can target alDH1L2 to regulate fatty acid oxidation (FAO), thereby inhibiting lipid accumulation and treating NAFLD. **(D)** In the progression of osteoporosis, ALDH1A1 can regulate NFATc1 expression, and disulfiram can also treat osteoporosis through the ALDH1A1-NFATc1 axis.

Parkinson’s disease (PD) is the most common degenerative movement disorder and primarily affects dopaminergic transmission in the basal ganglia. One of its most prominent pathological features is the preferential degeneration of dopaminergic neurons (DANs) in the ventral tier (Carmichael et al., 2021). The cytosolic ALDH1 enzyme family, which catalyzes aldehyde oxidation, plays a critical role in DANs. The accumulation of 3,4-dihydroxyphenylacetaldehyde (DOPAL), a toxic dopamine metabolite, contributes to neuronal damage. ALDH1A1 converts DOPAL into the less harmful 3,4-dihydroxyphenylacetic acid (DOPAC) (Figure 2A). Studies have demonstrated that reduced ALDH1A1 expression in DANs may contribute to PD pathogenesis, whereas elevated ALDH1A1 expression protects against dopaminergic neurodegeneration. Thus, targeting ALDH1A1 expression to reduce the abundance of toxic dopamine metabolites could serve as a preventive strategy for PD (Carmichael et al., 2021; Fan et al., 2022). Notably, research has revealed an association between ALDH1A1 genetic variants and PD susceptibility in the Han Chinese population. Key variants include the tag-SNP rs7043217 and haplotypes GGCTAG and GGTA. These findings provide genetic evidence for the role of ALDH1 in PD pathogenesis and could facilitate the development of screening strategies for PD risk alleles, thereby enabling early disease intervention (Fan et al., 2021). Furthermore, studies have confirmed that ALDH1A1 can regulate expression of the μ-opioid receptor and maintain the expression and activity of the μ-opioid receptor MOR1 through RA signaling, further modulating levodopa-induced motor dysfunction (Pan et al., 2019). Therefore, future research should further investigate the specific roles of ALDH1 isoforms (such as ALDH1A1/A2/A3) in PD pathogenesis and explore ALDH1-targeted neuroprotective therapeutic strategies.

### 4.2 ALDH1 family and cardiovascular diseases

Cardiovascular diseases (CVDs) remain the leading cause of mortality and disability worldwide. Early detection and treatment of CVDs are crucial for patient survival and long-term health. Despite advancements related to cardiovascular biomarkers, the prevalence of CVDs continues to rise worldwide, particularly with the aging of the global population (Kim et al., 2023). Research has confirmed that ALDH1 not only participates in alcohol metabolism but also plays a role in processes such as oxidative stress, apoptosis, and cell proliferation, all of which are closely related to CVDs. Atherosclerosis is one of the most common arteriosclerotic diseases. In M2 macrophages, the upregulation of ALDH1A2 expression and enzyme activity promotes the production of RA, inhibits inflammation, and slows the progression of atherosclerosis. Therefore, the ALDH1 family plays a protective role against atherosclerosis and helps slow its progression (Ouimet et al., 2015) (Figure 2B). Additionally, studies have shown that biallelic low-frequency variants in ALDH1A2 can lead to novel, lethal human congenital anomaly syndrome, including congenital diaphragmatic hernia and pulmonary and cardiovascular defects (Beecroft et al., 2021). Furthermore, studies utilizing adenovirus-mediated ALDH1A2 overexpression in primary cardiomyocytes have demonstrated successful ALDH1A2 upregulation and activation of the RA signaling pathway. Notably, ALDH1A2 overexpression not only increased cardiomyocyte size but also elevated the expression of embryonic-stage genes, suggesting a role in promoting cardiomyocyte hypertrophy and potential involvement in pathological cardiac remodeling (Liu et al., 2024). Future research should further elucidate whether ALDH1 expression functions solely as a biomarker or actively participates in the pathogenesis of various CVDs, including unexplored conditions such as pulmonary hypertension, heart failure, and arrhythmias. With continued investigation, ALDH1 may be established as a novel therapeutic target for cardiovascular diseases, offering more effective treatment strategies for patients.

### 4.3 ALDH1 and non-alcoholic fatty liver disease (NAFLD)

Nonalcoholic fatty liver disease (NAFLD), an inflammatory liver condition, can progress to nonalcoholic steatohepatitis (NASH), liver fibrosis, cirrhosis, and even hepatocellular carcinoma (Kennedy et al., 2021). NAFLD is characterized by excessive accumulation of neutral lipids (such as triglycerides) and hepatic steatosis. Among its pathogenic mechanisms, impaired fatty acid β-oxidation (FAO), a crucial biological process for fatty acid degradation, is closely associated with NAFLD progression. Studies have shown that hepatocyte FOXO1 can increase FAO by directly targeting ALDH1L2, thereby suppressing lipid deposition. Thus, modulating fatty acid oxidation via the FOXO1-ALDH1L2 axis may represent a potential therapeutic strategy for patients with NAFLD (Cheng et al., 2024) (Figure 2C). Additionally, research has demonstrated that ALDH1B1, as a transcriptional regulator of NR5A2, participates in pyroptosis by modulating reactive oxygen species (ROS) levels. Hepatocyte-specific NR5A2 expression can exacerbate NASH by downregulating ALDH1B1 expression and inducing pyroptosis (Zhao et al., 2024). Leveraging the tissue-specific distribution and functional features of these metabolic enzymes may offer opportunities for the effective prevention of chronic diseases. Furthermore, large-scale analysis of public -omics data has identified ALDH1A1 as a potential therapeutic target, providing a multitarget treatment strategy for future clinical research on NAFLD (Ma et al., 2021).

### 4.4 ALDH1 and osteoarthritis

Osteoarthritis (OA) is a highly prevalent and debilitating joint disorder for which no licensed therapies currently exist. The pathogenesis of OA is complex and involves genetic, mechanical, biochemical, and environmental factors. Among these factors, cartilage degradation is arguably the most critical driver of OA progression and is capable of activating both protective and inflammatory pathways within the tissue (Zhu and Vincent, 2023). Research has revealed that high-risk variants of the ALDH1A2 gene contribute to OA pathogenesis through multiple mechanisms. These variants not only reduce ALDH1A2 expression levels but also aberrantly activate inflammation-related signaling pathways. Notably, the enzyme encoded by ALDH1A2 catalyzes the synthesis of all-trans retinoic acid (ATRA), which can significantly mitigate cartilage damage by inducing protective gene expression (Duan et al., 2024). Additionally, research has shown that ALDH1A1 can regulate the expression of NFATc1, a key regulator of osteoclast differentiation, and that disulfiram can act through the ALDH1A1‒NFATc1 axis as a treatment for alcohol-related osteoporosis (Jia et al., 2019) (Figure 2D). These studies collectively suggest that the ALDH1 family represents a crucial potential therapeutic target for osteoarticular diseases and may lead to the development of novel targeted treatment strategies addressing mechanical inflammation in patients with OA and osteoporosis (Zhu et al., 2022).

## 5 Discussion

The mechanistic roles of ALDH1 in various diseases are gradually being revealed. Research has confirmed that the ALDH1 family not only participates in multiple pathophysiological processes—such as aldehyde detoxification, RA signaling pathways, and cancer cell proliferation, invasion, and migration—but is also closely associated with tumor growth, metastasis, and prognosis in patients with cancers. Discoveries regarding the mechanisms of ALDH1 in nonneoplastic diseases have established this family of enzymes as a highly promising therapeutic target for conditions such as NAFLD, PD, OA, and CVDs, where its expression downregulation primarily leads to the loss of protective functions.

Current research shortcomings include the following: despite the established involvement of ALDH1 in key pathways, such as aldehyde clearance, RA signaling, stem cell maintenance, and metabolic regulation, investigations have remained narrowly focused on single isoforms. Moreover, disease association studies show significant bias, with approximately 80% of the literature concentrated on oncology; however, substantial gaps regarding nonneoplastic and metabolic diseases remain, and studies rarely explore interisoform interactions within shared pathological contexts. Finally, although inhibitor development has progressed, it remains constrained by inadequate subtype selectivity, whereas agonist design for nonneoplastic conditions remains virtually unexplored. Additionally, fundamental knowledge gaps persist regarding ALDH1 family dynamics in terms of subcellular localization, posttranslational modification regulation, and epigenetic coordination networks. Consequently, future ALDH1-targeted therapeutic development should prioritize creating subtype-selective inhibitors for oncology and activity-enhancing agents for other diseases while exploring combination regimens with conventional and multitarget therapies. Continued investigations into the mechanisms of ALDH1 in nonneoplastic diseases are essential to fully elucidate its functions beyond oncology. Through persistent exploration, ALDH1-targeted therapies are poised to play increasingly pivotal roles across disease research domains, ultimately achieving transformative breakthroughs from bench to bedside and delivering paradigm-shifting treatment strategies for diverse pathologies, including cancer.
